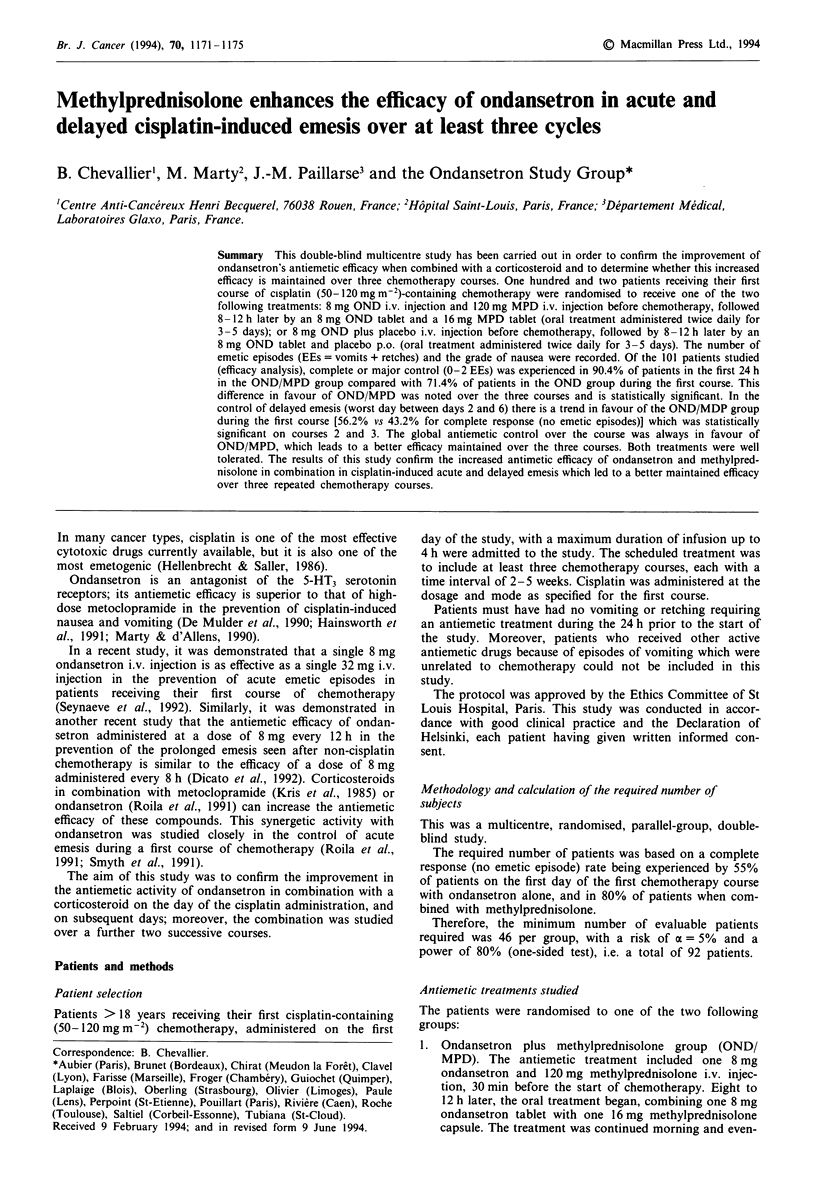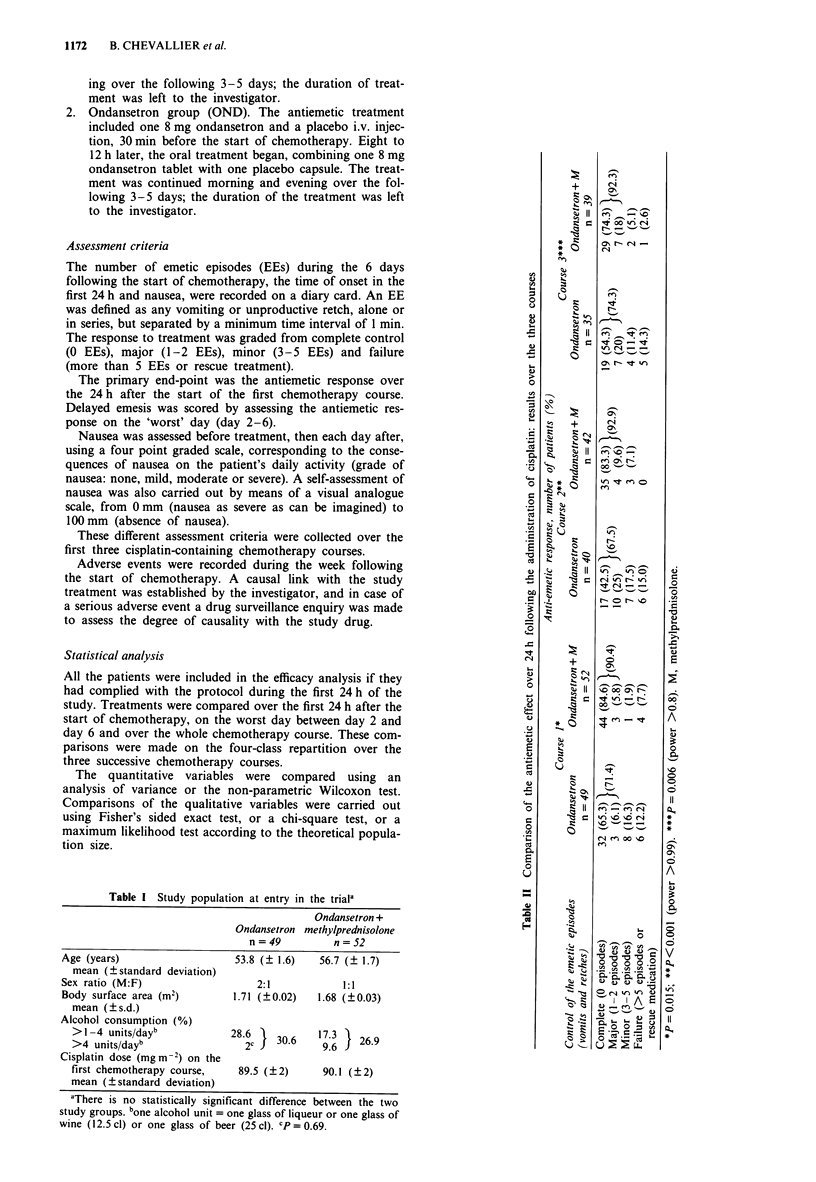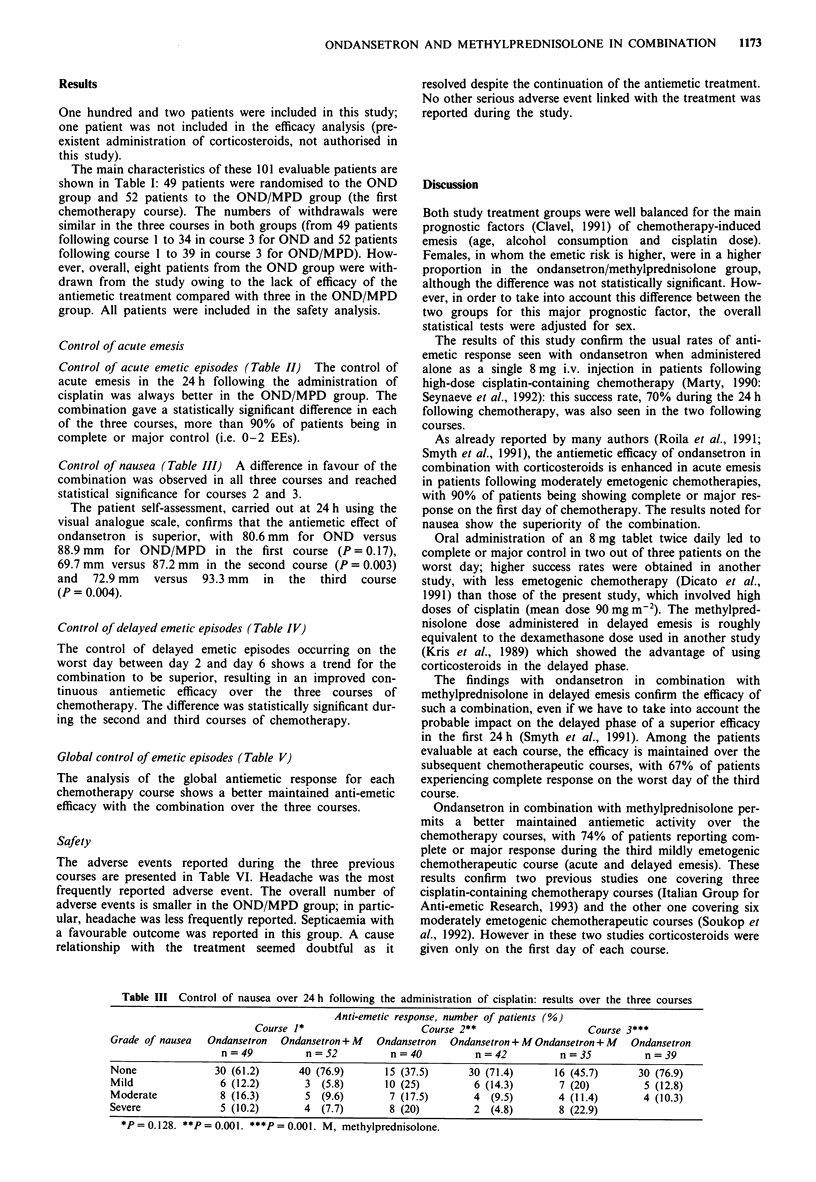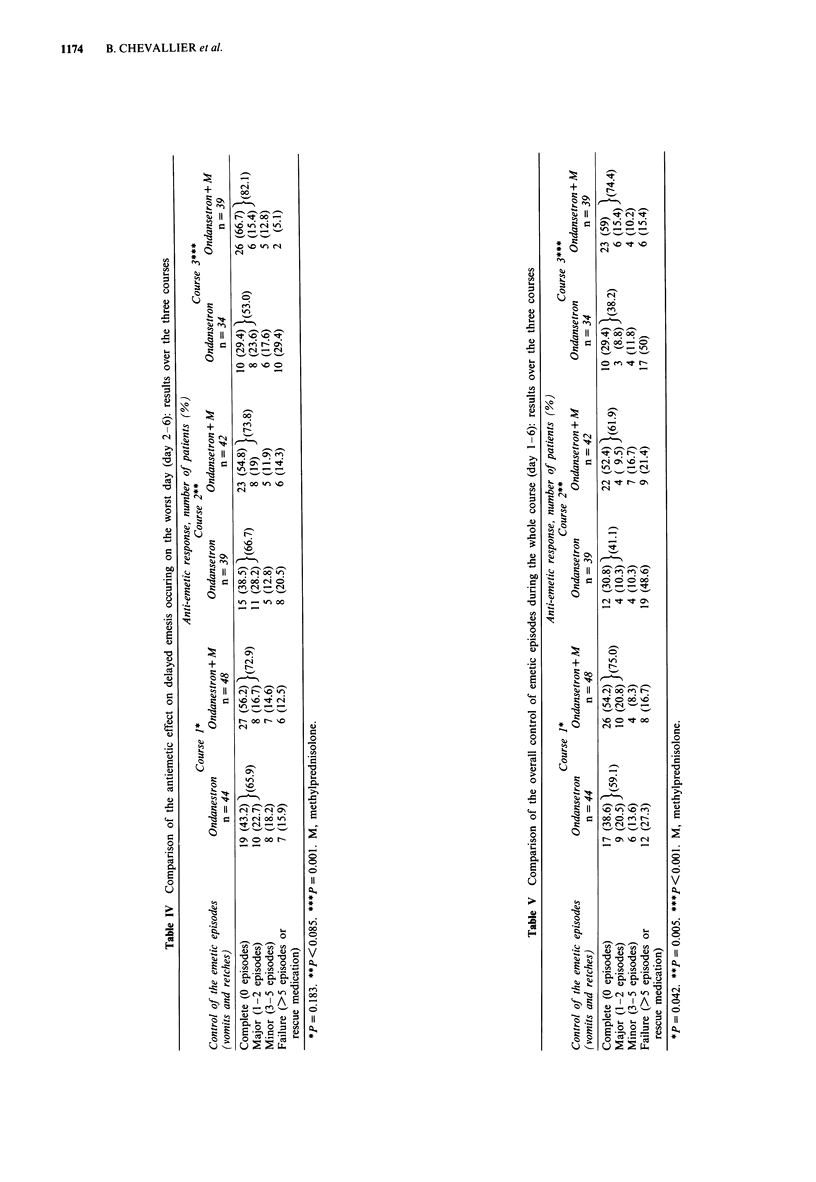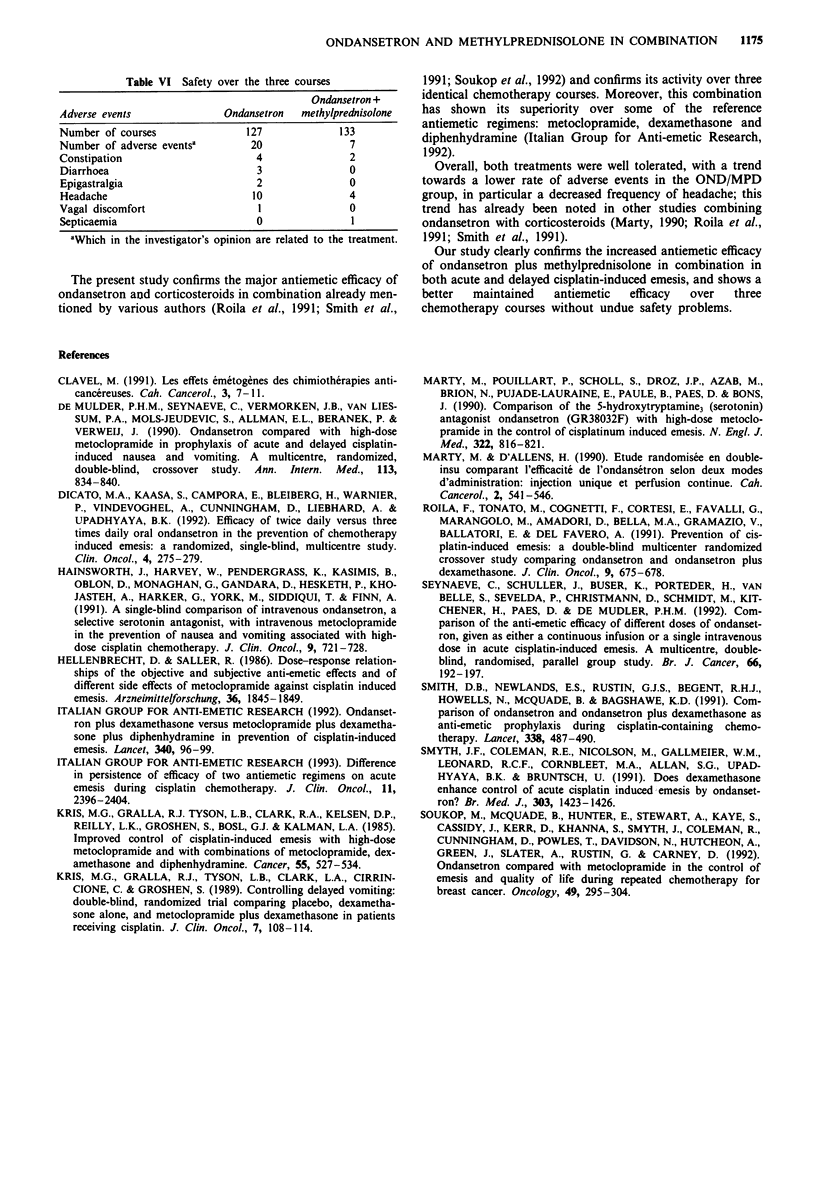# Methylprednisolone enhances the efficacy of ondansetron in acute and delayed cisplatin-induced emesis over at least three cycles. Ondansetron Study Group.

**DOI:** 10.1038/bjc.1994.467

**Published:** 1994-12

**Authors:** B. Chevallier, M. Marty, J. M. Paillarse

**Affiliations:** Centre Anti-Cancéreux Henri Becquerel, Rouen, France.

## Abstract

This double-blind multicentre study has been carried out in order to confirm the improvement of ondansetron's antiemetic efficacy when combined with a corticosteroid and to determine whether this increased efficacy is maintained over three chemotherapy courses. One hundred and two patients receiving their first course of cisplatin (50-120 mg m-2)-containing chemotherapy were randomised to receive one of the two following treatments: 8 mg OND i.v. injection and 120 mg MPD i.v. injection before chemotherapy, followed 8-12 h later by an 8 mg OND tablet and a 16 mg MPD tablet (oral treatment administered twice daily for 3-5 days): or 8 mg OND plus placebo i.v. injection before chemotherapy, followed by 8-12 h later by an 8 mg OND tablet and placebo p.o. (oral treatment administered twice daily for 3-5 days). The number of emetic episodes (EEs = vomits + retches) and the grade of nausea were recorded. Of the 101 patients studied (efficacy analysis), complete or major control (0-2 EEs) was experienced in 90.4% of patients in the first 24 h in the OND/MPD group compared with 71.4% of patients in the OND group during the first course. This difference in favour of OND/MPD was noted over the three courses and is statistically significant. In the control of delayed emesis (worst day between days 2 and 6) there is a trend in favour of the OND/MDP group during the first course [56.2% vs 43.2% for complete response (no emetic episodes)] which was statistically significant on courses 2 and 3. The global antiemetic control over the course was always in favour of OND/MPD, which leads to a better efficacy maintained over the three courses. Both treatments were well tolerated. The results of this study confirm the increased antimetic efficacy of ondansetron and methylprednisolone in combination in cisplatin-induced acute and delayed emesis which led to a better maintained efficacy over three repeated chemotherapy courses.


Br. J. Cancer (1994), 70, 1171–1175

© Macmillan Press Ltd., 1994

### Methylprednisolone enhances the efficacy of ondansetron in acute and delayed cisplatin-induced emesis over at least three cycles

B. Chevallier[1], M. Marty[2], J.-M. Paillarse[3] and the Ondansetron Study Group*

[1]Centre Anti-Cancéreux Henri Becquerel, 76038 Rouen, France; [2]Hôpital Saint-Louis, Paris, France; [3]Département Médical, Laboratoires Glaxo, Paris, France.

**Summary** This double-blind multicentre study has been carried out in order to confirm the improvement of ondansetron's antiemetic efficacy when combined with a corticosteroid and to determine whether this increased efficacy is maintained over three chemotherapy courses. One hundred and two patients receiving their first course of cisplatin (50–120 mg m$^{-2}$)-containing chemotherapy were randomised to receive one of the two following treatments: 8 mg OND i.v. injection and 120 mg MPD i.v. injection before chemotherapy, followed 8–12 h later by an 8 mg OND tablet and a 16 mg MPD tablet (oral treatment administered twice daily for 3–5 days); or 8 mg OND plus placebo i.v. injection before chemotherapy, followed by 8–12 h later by an 8 mg OND tablet and placebo p.o. (oral treatment administered twice daily for 3–5 days). The number of emetic episodes (EEs = vomits + retches) and the grade of nausea were recorded. Of the 101 patients studied (efficacy analysis), complete or major control (0–2 EEs) was experienced in 90.4% of patients in the first 24 h in the OND/MPD group compared with 71.4% of patients in the OND group during the first course. This difference in favour of OND/MPD was noted over the three courses and is statistically significant. In the control of delayed emesis (worst day between days 2 and 6) there is a trend in favour of the OND/MDP group during the first course [56.2% *vs* 43.2% for complete response (no emetic episodes)] which was statistically significant on courses 2 and 3. The global antiemetic control over the course was always in favour of OND/MPD, which leads to a better efficacy maintained over the three courses. Both treatments were well tolerated. The results of this study confirm the increased antimetic efficacy of ondansetron and methylprednisolone in combination in cisplatin-induced acute and delayed emesis which led to a better maintained efficacy over three repeated chemotherapy courses.

In many cancer types, cisplatin is one of the most effective cytotoxic drugs currently available, but it is also one of the most emetogenic (Hellenbrecht & Saller, 1986).

Ondansetron is an antagonist of the 5-$HT_3$ serotonin receptors; its antiemetic efficacy is superior to that of high-dose metoclopramide in the prevention of cisplatin-induced nausea and vomiting (De Mulder *et al.*, 1990; Hainsworth *et al.*, 1991; Marty & d'Allens, 1990).

In a recent study, it was demonstrated that a single 8 mg ondansetron i.v. injection is as effective as a single 32 mg i.v. injection in the prevention of acute emetic episodes in patients receiving their first course of chemotherapy (Seynaeve *et al.*, 1992). Similarly, it was demonstrated in another recent study that the antiemetic efficacy of ondansetron administered at a dose of 8 mg every 12 h in the prevention of the prolonged emesis seen after non-cisplatin chemotherapy is similar to the efficacy of a dose of 8 mg administered every 8 h (Dicato *et al.*, 1992). Corticosteroids in combination with metoclopramide (Kris *et al.*, 1985) or ondansetron (Roila *et al.*, 1991) can increase the antiemetic efficacy of these compounds. This synergetic activity with ondansetron was studied closely in the control of acute emesis during a first course of chemotherapy (Roila *et al.*, 1991; Smyth *et al.*, 1991).

The aim of this study was to confirm the improvement in the antiemetic activity of ondansetron in combination with a corticosteroid on the day of the cisplatin administration, and on subsequent days; moreover, the combination was studied over a further two successive courses.

### Patients and methods

### *Patient selection*

Patients >18 years receiving their first cisplatin-containing (50–120 mg m$^{-2}$) chemotherapy, administered on the first day of the study, with a maximum duration of infusion up to 4 h were admitted to the study. The scheduled treatment was to include at least three chemotherapy courses, each with a time interval of 2–5 weeks. Cisplatin was administered at the dosage and mode as specified for the first course.

Patients must have had no vomiting or retching requiring an antiemetic treatment during the 24 h prior to the start of the study. Moreover, patients who received other active antiemetic drugs because of episodes of vomiting which were unrelated to chemotherapy could not be included in this study.

The protocol was approved by the Ethics Committee of St Louis Hospital, Paris. This study was conducted in accordance with good clinical practice and the Declaration of Helsinki, each patient having given written informed consent.

### *Methodology and calculation of the required number of subjects*

This was a multicentre, randomised, parallel-group, double-blind study.

The required number of patients was based on a complete response (no emetic episode) rate being experienced by 55% of patients on the first day of the first chemotherapy course with ondansetron alone, and in 80% of patients when combined with methylprednisolone.

Therefore, the minimum number of evaluable patients required was 46 per group, with a risk of $\alpha = 5\%$ and a power of 80% (one-sided test), i.e. a total of 92 patients.

### *Antiemetic treatments studied*

The patients were randomised to one of the two following groups:

1. Ondansetron plus methylprednisolone group (OND/MPD). The antiemetic treatment included one 8 mg ondansetron and 120 mg methylprednisolone i.v. injection, 30 min before the start of chemotherapy. Eight to 12 h later, the oral treatment began, combining one 8 mg ondansetron tablet with one 16 mg methylprednisolone capsule. The treatment was continued morning and even-

Correspondence: B. Chevallier.

*Aubier (Paris), Brunet (Bordeaux), Chirat (Meudon la Forêt), Clavel (Lyon), Farisse (Marseille), Froger (Chambéry), Guiochet (Quimper), Laplaige (Blois), Oberling (Strasbourg), Olivier (Limoges), Paule (Lens), Perpoint (St-Etienne), Pouillart (Paris), Rivière (Caen), Roche (Toulouse), Saltiel (Corbeil-Essonne), Tubiana (St-Cloud).

Received 9 February 1994; and in revised form 9 June 1994.

ing over the following 3–5 days; the duration of treatment was left to the investigator.

2. Ondansetron group (OND). The antiemetic treatment included one 8 mg ondansetron and a placebo i.v. injection, 30 min before the start of chemotherapy. Eight to 12 h later, the oral treatment began, combining one 8 mg ondansetron tablet with one placebo capsule. The treatment was continued morning and evening over the following 3–5 days; the duration of the treatment was left to the investigator.

### *Assessment criteria*

The number of emetic episodes (EEs) during the 6 days following the start of chemotherapy, the time of onset in the first 24 h and nausea, were recorded on a diary card. An EE was defined as any vomiting or unproductive retch, alone or in series, but separated by a minimum time interval of 1 min. The response to treatment was graded from complete control (0 EEs), major (1–2 EEs), minor (3–5 EEs) and failure (more than 5 EEs or rescue treatment).

The primary end-point was the antiemetic response over the 24 h after the start of the first chemotherapy course. Delayed emesis was scored by assessing the antiemetic response on the 'worst' day (day 2–6).

Nausea was assessed before treatment, then each day after, using a four point graded scale, corresponding to the consequences of nausea on the patient's daily activity (grade of nausea: none, mild, moderate or severe). A self-assessment of nausea was also carried out by means of a visual analogue scale, from 0 mm (nausea as severe as can be imagined) to 100 mm (absence of nausea).

These different assessment criteria were collected over the first three cisplatin-containing chemotherapy courses.

Adverse events were recorded during the week following the start of chemotherapy. A causal link with the study treatment was established by the investigator, and in case of a serious adverse event a drug surveillance enquiry was made to assess the degree of causality with the study drug.

### *Statistical analysis*

All the patients were included in the efficacy analysis if they had complied with the protocol during the first 24 h of the study. Treatments were compared over the first 24 h after the start of chemotherapy, on the worst day between day 2 and day 6 and over the whole chemotherapy course. These comparisons were made on the four-class repartition over the three successive chemotherapy courses.

The quantitative variables were compared using an analysis of variance or the non-parametric Wilcoxon test. Comparisons of the qualitative variables were carried out using Fisher's sided exact test, or a chi-square test, or a maximum likelihood test according to the theoretical population size.

**Table I** Study population at entry in the trial[a]

| | *Ondansetron* n = *49* | *Ondansetron + methylprednisolone* *n* = *52* |
|---|---|---|
| Age (years) mean (± standard deviation) | 53.8 (± 1.6) | 56.7 (± 1.7) |
| Sex ratio (M:F) | 2:1 | 1:1 |
| Body surface area ($m^2$) mean (± s.d.) | 1.71 (± 0.02) | 1.68 (± 0.03) |
| Alcohol consumption (%) | | |
| >1–4 units/day[b] | 28.6 } 30.6 | 17.3 } 26.9 |
| >4 units/day[b] | 2[c] | 9.6 |
| Cisplatin dose (mg $m^{-2}$) on the first chemotherapy course, mean (± standard deviation) | 89.5 (± 2) | 90.1 (± 2) |

[a]There is no statistically significant difference between the two study groups. [b]one alcohol unit = one glass of liqueur or one glass of wine (12.5 cl) or one glass of beer (25 cl). [c]$P = 0.69$.

**Table II** Comparison of the antiemetic effect over 24 h following the administration of cisplatin: results over the three courses

| *Control of the emetic episodes (vomits and retches)* | *Anti-emetic response, number of patients (%)* Course 1* *Ondansetron* n = *49* | Course 1* *Ondansetron + M* n = *52* | Course 2** *Ondansetron* n = *40* | Course 2** *Ondansetron + M* n = *42* | Course 3*** *Ondansetron* n = *35* | Course 3*** *Ondansetron + M* n = *39* |
|---|---|---|---|---|---|---|
| Complete (0 episodes) | 32 (65.3) } (71.4) | 44 (84.6) } (90.4) | 17 (42.5) } (67.5) | 35 (83.3) } (92.9) | 19 (54.3) } (74.3) | 29 (74.3) } (92.3) |
| Major (1–2 episodes) | 3 (6.1) | 3 (5.8) | 10 (25) | 4 (9.6) | 7 (20) | 7 (18) |
| Minor (3–5 episodes) | 8 (16.3) | 1 (1.9) | 7 (17.5) | 3 (7.1) | 4 (11.4) | 2 (5.1) |
| Failure (>5 episodes or rescue medication) | 6 (12.2) | 4 (7.7) | 6 (15.0) | 0 | 5 (14.3) | 1 (2.6) |

*$P = 0.015$; **$P < 0.001$ (power >0.99). ***$P = 0.006$ (power >0.8). M, methylprednisolone.

### Results

One hundred and two patients were included in this study; one patient was not included in the efficacy analysis (pre-existent administration of corticosteroids, not authorised in this study).

The main characteristics of these 101 evaluable patients are shown in Table I: 49 patients were randomised to the OND group and 52 patients to the OND/MPD group (the first chemotherapy course). The numbers of withdrawals were similar in the three courses in both groups (from 49 patients following course 1 to 34 in course 3 for OND and 52 patients following course 1 to 39 in course 3 for OND/MPD). However, overall, eight patients from the OND group were withdrawn from the study owing to the lack of efficacy of the antiemetic treatment compared with three in the OND/MPD group. All patients were included in the safety analysis.

### *Control of acute emesis*

*Control of acute emetic episodes (Table II)* The control of acute emesis in the 24 h following the administration of cisplatin was always better in the OND/MPD group. The combination gave a statistically significant difference in each of the three courses, more than 90% of patients being in complete or major control (i.e. 0–2 EEs).

*Control of nausea (Table III)* A difference in favour of the combination was observed in all three courses and reached statistical significance for courses 2 and 3.

The patient self-assessment, carried out at 24 h using the visual analogue scale, confirms that the antiemetic effect of ondansetron is superior, with 80.6 mm for OND versus 88.9 mm for OND/MPD in the first course ($P = 0.17$), 69.7 mm versus 87.2 mm in the second course ($P = 0.003$) and 72.9 mm versus 93.3 mm in the third course ($P = 0.004$).

### *Control of delayed emetic episodes (Table IV)*

The control of delayed emetic episodes occurring on the worst day between day 2 and day 6 shows a trend for the combination to be superior, resulting in an improved continuous antiemetic efficacy over the three courses of chemotherapy. The difference was statistically significant during the second and third courses of chemotherapy.

### *Global control of emetic episodes (Table V)*

The analysis of the global antiemetic response for each chemotherapy course shows a better maintained anti-emetic efficacy with the combination over the three courses.

### *Safety*

The adverse events reported during the three previous courses are presented in Table VI. Headache was the most frequently reported adverse event. The overall number of adverse events is smaller in the OND/MPD group; in particular, headache was less frequently reported. Septicaemia with a favourable outcome was reported in this group. A cause relationship with the treatment seemed doubtful as it resolved despite the continuation of the antiemetic treatment. No other serious adverse event linked with the treatment was reported during the study.

### Discussion

Both study treatment groups were well balanced for the main prognostic factors (Clavel, 1991) of chemotherapy-induced emesis (age, alcohol consumption and cisplatin dose). Females, in whom the emetic risk is higher, were in a higher proportion in the ondansetron/methylprednisolone group, although the difference was not statistically significant. However, in order to take into account this difference between the two groups for this major prognostic factor, the overall statistical tests were adjusted for sex.

The results of this study confirm the usual rates of antiemetic response seen with ondansetron when administered alone as a single 8 mg i.v. injection in patients following high-dose cisplatin-containing chemotherapy (Marty, 1990: Seynaeve *et al.*, 1992): this success rate, 70% during the 24 h following chemotherapy, was also seen in the two following courses.

As already reported by many authors (Roila *et al.*, 1991; Smyth *et al.*, 1991), the antiemetic efficacy of ondansetron in combination with corticosteroids is enhanced in acute emesis in patients following moderately emetogenic chemotherapies, with 90% of patients being showing complete or major response on the first day of chemotherapy. The results noted for nausea show the superiority of the combination.

Oral administration of an 8 mg tablet twice daily led to complete or major control in two out of three patients on the worst day; higher success rates were obtained in another study, with less emetogenic chemotherapy (Dicato *et al.*, 1991) than those of the present study, which involved high doses of cisplatin (mean dose 90 mg $m^{-2}$). The methylprednisolone dose administered in delayed emesis is roughly equivalent to the dexamethasone dose used in another study (Kris *et al.*, 1989) which showed the advantage of using corticosteroids in the delayed phase.

The findings with ondansetron in combination with methylprednisolone in delayed emesis confirm the efficacy of such a combination, even if we have to take into account the probable impact on the delayed phase of a superior efficacy in the first 24 h (Smyth *et al.*, 1991). Among the patients evaluable at each course, the efficacy is maintained over the subsequent chemotherapeutic courses, with 67% of patients experiencing complete response on the worst day of the third course.

Ondansetron in combination with methylprednisolone permits a better maintained antiemetic activity over the chemotherapy courses, with 74% of patients reporting complete or major response during the third mildly emetogenic chemotherapeutic course (acute and delayed emesis). These results confirm two previous studies one covering three cisplatin-containing chemotherapy courses (Italian Group for Anti-emetic Research, 1993) and the other one covering six moderately emetogenic chemotherapeutic courses (Soukop *et al.*, 1992). However in these two studies corticosteroids were given only on the first day of each course.

**Table III** Control of nausea over 24 h following the administration of cisplatin: results over the three courses

| | *Anti-emetic response, number of patients (%)* | | | | | |
|---|---|---|---|---|---|---|
| | *Course 1** | | *Course 2*** | | *Course 3**** | |
| *Grade of nausea* | *Ondansetron* n = *49* | *Ondansetron + M* n = *52* | *Ondansetron* n = *40* | *Ondansetron + M* n = *42* | *Ondansetron + M* n = *35* | *Ondansetron* n = *39* |
| None | 30 (61.2) | 40 (76.9) | 15 (37.5) | 30 (71.4) | 16 (45.7) | 30 (76.9) |
| Mild | 6 (12.2) | 3 (5.8) | 10 (25) | 6 (14.3) | 7 (20) | 5 (12.8) |
| Moderate | 8 (16.3) | 5 (9.6) | 7 (17.5) | 4 (9.5) | 4 (11.4) | 4 (10.3) |
| Severe | 5 (10.2) | 4 (7.7) | 8 (20) | 2 (4.8) | 8 (22.9) | |

*$P = 0.128$. **$P = 0.001$. ***$P = 0.001$. M, methylprednisolone.

**Table IV** Comparison of the antiemetic effect on delayed emesis occuring on the worst day (day 2–6): results over the three courses

| | Anti-emetic response, number of patients (%) | | | | | |
|---|---|---|---|---|---|---|
| | *Course 1** | | *Course 2*** | | *Course 3**** | |
| *Control of the emetic episodes (vomits and retches)* | *Ondanestron* n = *44* | *Ondanestron + M* n = *48* | *Ondansetron* n = *39* | *Ondansetron + M* n = *42* | *Ondansetron* n = *34* | *Ondansetron + M* n = *39* |
| Complete (0 episodes) | 19 (43.2) } (65.9) | 27 (56.2) } (72.9) | 15 (38.5) } (66.7) | 23 (54.8) } (73.8) | 10 (29.4) } (53.0) | 26 (66.7) } (82.1) |
| Major (1–2 episodes) | 10 (22.7) } (65.9) | 8 (16.7) } (72.9) | 11 (28.2) } (66.7) | 8 (19) } (73.8) | 8 (23.6) } (53.0) | 6 (15.4) } (82.1) |
| Minor (3–5 episodes) | 8 (18.2) | 7 (14.6) | 5 (12.8) | 5 (11.9) | 6 (17.6) | 5 (12.8) |
| Failure (>5 episodes or rescue medication) | 7 (15.9) | 6 (12.5) | 8 (20.5) | 6 (14.3) | 10 (29.4) | 2 (5.1) |

$^{*}P = 0.183$. $^{**}P < 0.085$. $^{***}P = 0.001$. M, methylprednisolone.

**Table V** Comparison of the overall control of emetic episodes during the whole course (day 1–6): results over the three courses

| | Anti-emetic response, number of patients (%) | | | | | |
|---|---|---|---|---|---|---|
| | *Course 1** | | *Course 2*** | | *Course 3**** | |
| *Control of the emetic episodes (vomits and retches)* | *Ondansetron* n = *44* | *Ondansetron + M* n = *48* | *Ondansetron* n = *39* | *Ondansetron + M* n = *42* | *Ondansetron* n = *34* | *Ondansetron + M* n = *39* |
| Complete (0 episodes) | 17 (38.6) } (59.1) | 26 (54.2) } (75.0) | 12 (30.8) } (41.1) | 22 (52.4) } (61.9) | 10 (29.4) } (38.2) | 23 (59) } (74.4) |
| Major (1–2 episodes) | 9 (20.5) } (59.1) | 10 (20.8) } (75.0) | 4 (10.3) } (41.1) | 4 ( 9.5) } (61.9) | 3 (8.8) } (38.2) | 6 (15.4) } (74.4) |
| Minor (3–5 episodes) | 6 (13.6) | 4 (8.3) | 4 (10.3) | 7 (16.7) | 4 (11.8) | 4 (10.2) |
| Failure (>5 episodes or rescue medication) | 12 (27.3) | 8 (16.7) | 19 (48.6) | 9 (21.4) | 17 (50) | 6 (15.4) |

$^{*}P = 0.042$. $^{**}P = 0.005$. $^{***}P < 0.001$. M, methylprednisolone.

**Table VI** Safety over the three courses

| *Adverse events* | *Ondansetron* | *Ondansetron+ methylprednisolone* |
|---|---|---|
| Number of courses | 127 | 133 |
| Number of adverse events[a] | 20 | 7 |
| Constipation | 4 | 2 |
| Diarrhoea | 3 | 0 |
| Epigastralgia | 2 | 0 |
| Headache | 10 | 4 |
| Vagal discomfort | 1 | 0 |
| Septicaemia | 0 | 1 |

[a]Which in the investigator's opinion are related to the treatment.

The present study confirms the major antiemetic efficacy of ondansetron and corticosteroids in combination already mentioned by various authors (Roila *et al.*, 1991; Smith *et al.*, 1991; Soukop *et al.*, 1992) and confirms its activity over three identical chemotherapy courses. Moreover, this combination has shown its superiority over some of the reference antiemetic regimens: metoclopramide, dexamethasone and diphenhydramine (Italian Group for Anti-emetic Research, 1992).

Overall, both treatments were well tolerated, with a trend towards a lower rate of adverse events in the OND/MPD group, in particular a decreased frequency of headache; this trend has already been noted in other studies combining ondansetron with corticosteroids (Marty, 1990; Roila *et al.*, 1991; Smith *et al.*, 1991).

Our study clearly confirms the increased antiemetic efficacy of ondansetron plus methylprednisolone in combination in both acute and delayed cisplatin-induced emesis, and shows a better maintained antiemetic efficacy over three chemotherapy courses without undue safety problems.

### References

CLAVEL, M. (1991). Les effets émétogènes des chimiothérapies anticancéreuses. *Cah. Cancerol.*, **3**, 7-11.

DE MULDER, P.H.M., SEYNAEVE, C., VERMORKEN, J.B., VAN LIESSUM, P.A., MOLS-JEUDEVIC, S., ALLMAN, E.L., BERANEK, P. & VERWEIJ, J. (1990). Ondansetron compared with high-dose metoclopramide in prophylaxis of acute and delayed cisplatin-induced nausea and vomiting. A multicentre, randomized, double-blind, crossover study. *Ann. Intern. Med.*, **113**, 834-840.

DICATO, M.A., KAASA, S., CAMPORA, E., BLEIBERG, H., WARNIER, P., VINDEVOGHEL, A., CUNNINGHAM, D., LIEBHARD, A. & UPADHYAYA, B.K. (1992). Efficacy of twice daily versus three times daily oral ondansetron in the prevention of chemotherapy induced emesis: a randomized, single-blind, multicentre study. *Clin. Oncol.*, **4**, 275-279.

HAINSWORTH, J., HARVEY, W., PENDERGRASS, K., KASIMIS, B., OBLON, D., MONAGHAN, G., GANDARA, D., HESKETH, P., KHOJASTEH, A., HARKER, G., YORK, M., SIDDIQUI, T. & FINN, A. (1991). A single-blind comparison of intravenous ondansetron, a selective serotonin antagonist, with intravenous metoclopramide in the prevention of nausea and vomiting associated with high-dose cisplatin chemotherapy. *J. Clin. Oncol.*, **9**, 721-728.

HELLENBRECHT, D. & SALLER, R. (1986). Dose–response relationships of the objective and subjective anti-emetic effects and of different side effects of metoclopramide against cisplatin induced emesis. *Arzneimittelforschung*, **36**, 1845-1849.

ITALIAN GROUP FOR ANTI-EMETIC RESEARCH (1992). Ondansetron plus dexamethasone versus metoclopramide plus dexamethasone plus diphenhydramine in prevention of cisplatin-induced emesis. *Lancet*, **340**, 96-99.

ITALIAN GROUP FOR ANTI-EMETIC RESEARCH (1993). Difference in persistence of efficacy of two antiemetic regimens on acute emesis during cisplatin chemotherapy. *J. Clin. Oncol.*, **11**, 2396-2404.

KRIS, M.G., GRALLA, R.J. TYSON, L.B., CLARK, R.A., KELSEN, D.P., REILLY, L.K., GROSHEN, S., BOSL, G.J. & KALMAN, L.A. (1985). Improved control of cisplatin-induced emesis with high-dose metoclopramide and with combinations of metoclopramide, dexamethasone and diphenhydramine. *Cancer*, **55**, 527-534.

KRIS, M.G., GRALLA, R.J., TYSON, L.B., CLARK, L.A., CIRRINCIONE, C. & GROSHEN, S. (1989). Controlling delayed vomiting: double-blind, randomized trial comparing placebo, dexamethasone alone, and metoclopramide plus dexamethasone in patients receiving cisplatin. *J. Clin. Oncol.*, **7**, 108-114.

MARTY, M., POUILLART, P., SCHOLL, S., DROZ, J.P., AZAB, M., BRION, N., PUJADE-LAURAINE, E., PAULE, B., PAES, D. & BONS, J. (1990). Comparison of the 5-hydroxytryptamine$_3$ (serotonin) antagonist ondansetron (GR38032F) with high-dose metoclopramide in the control of cisplatinum induced emesis. *N. Engl. J. Med.*, **322**, 816-821.

MARTY, M. & D'ALLENS, H. (1990). Etude randomisée en double-insu comparant l'efficacité de l'ondansétron selon deux modes d'administration: injection unique et perfusion continue. *Cah. Cancerol.*, **2**, 541-546.

ROILA, F., TONATO, M., COGNETTI, F., CORTESI, E., FAVALLI, G., MARANGOLO, M., AMADORI, D., BELLA, M.A., GRAMAZIO, V., BALLATORI, E. & DEL FAVERO, A. (1991). Prevention of cisplatin-induced emesis: a double-blind multicenter randomized crossover study comparing ondansetron and ondansetron plus dexamethasone. *J. Clin. Oncol.*, **9**, 675-678.

SEYNAEVE, C., SCHULLER, J., BUSER, K., PORTEDER, H., VAN BELLE, S., SEVELDA, P., CHRISTMANN, D., SCHMIDT, M., KITCHENER, H., PAES, D. & DE MUDLER, P.H.M. (1992). Comparison of the anti-emetic efficacy of different doses of ondansetron, given as either a continuous infusion or a single intravenous dose in acute cisplatin-induced emesis. A multicentre, double-blind, randomised, parallel group study. *Br. J. Cancer*, **66**, 192-197.

SMITH, D.B., NEWLANDS, E.S., RUSTIN, G.J.S., BEGENT, R.H.J., HOWELLS, N., MCQUADE, B. & BAGSHAWE, K.D. (1991). Comparison of ondansetron and ondansetron plus dexamethasone as anti-emetic prophylaxis during cisplatin-containing chemotherapy. *Lancet*, **338**, 487-490.

SMYTH, J.F., COLEMAN, R.E., NICOLSON, M., GALLMEIER, W.M., LEONARD, R.C.F., CORNBLEET, M.A., ALLAN, S.G., UPADHYAYA, B.K. & BRUNTSCH, U. (1991). Does dexamethasone enhance control of acute cisplatin induced emesis by ondansetron? *Br. Med. J.*, **303**, 1423-1426.

SOUKOP, M., MCQUADE, B., HUNTER, E., STEWART, A., KAYE, S., CASSIDY, J., KERR, D., KHANNA, S., SMYTH, J., COLEMAN, R., CUNNINGHAM, D., POWLES, T., DAVIDSON, N., HUTCHEON, A., GREEN, J., SLATER, A., RUSTIN, G. & CARNEY, D. (1992). Ondansetron compared with metoclopramide in the control of emesis and quality of life during repeated chemotherapy for breast cancer. *Oncology*, **49**, 295-304.